# Implementation of Telehealth Services to Assess, Monitor, and Treat Neurodevelopmental Disorders: Systematic Review

**DOI:** 10.2196/22619

**Published:** 2021-01-20

**Authors:** Althea Z Valentine, Sophie S Hall, Emma Young, Beverley J Brown, Madeleine J Groom, Chris Hollis, Charlotte L Hall

**Affiliations:** 1 Institute of Mental Health School of Medicine, Division of Psychiatry and Applied Psychology University of Nottingham Nottingham United Kingdom; 2 Department of Neuroscience, Psychology and Behaviour University of Leicester Centre for Medicine Leicester United Kingdom; 3 Nottinghamshire Healthcare National Health Service Foundation Trust Library and Knowledge Services Duncan Macmillan House Staff Library Nottingham United Kingdom; 4 National Institute for Health Research MindTech MedTech Co-operative Institute of Mental Health, School of Medicine, Division of Psychiatry and Applied Psychology University of Nottingham Nottingham United Kingdom; 5 Department of Child and Adolescent Psychiatry Queen's Medical Centre Nottingham United Kingdom

**Keywords:** neurodevelopmental disorders, technology, telehealth, review, COVID-19, implement, effective, mental health

## Abstract

**Background:**

In response to COVID-19, there has been increasing momentum in telehealth development and delivery. To assess the anticipated exponential growth in telehealth, it is important to accurately capture how telehealth has been used in specific mental health fields prior to the pandemic.

**Objective:**

This systematic review aimed to highlight how telehealth has been used with clinical samples in the neurodevelopmental field, including patients with neurodevelopmental disorders (NDDs), their families, and health care professionals. To identify which technologies show the greatest potential for implementation into health services, we evaluated technologies for effectiveness, economic impact, and readiness for clinical adoption.

**Methods:**

A systematic search of literature was undertaken in April 2018 and updated until December 2019, by using the Medline, Web of Science, Scopus, CINAHL Plus, EMBASE, and PsycInfo databases. Extracted data included the type of technology, how the technology was used (ie, assessment, treatment, and monitoring), participant characteristics, reported outcomes and authors’ views on clinical effectiveness, user impact (ie, feasibility and acceptability), economic impact, and readiness for clinic adoption. A quality review of the research was performed in accordance with the Oxford Centre for Evidence-Based Medicine Levels of Evidence.

**Results:**

A total of 42 studies met the inclusion criteria. These studies included participants and family members with autism spectrum disorders (21/42, 50%), attention deficit hyperactivity disorders (8/42, 19%), attention deficit hyperactivity or autism spectrum disorders (3/42, 7%), communication disorders (7/42, 17%), and tic disorders (2/42, 5%). The focus of most studies (33/42, 79%) was on treatment, rather than assessment (4/42, 10%) or monitoring (5/42, 12%). Telehealth services demonstrated promise for being clinically effective, predominantly in relation to diagnosing and monitoring NDDs. In terms of NDD treatment, telehealth services were usually equivalent to control groups. There was some evidence of positive user and economic impacts, including increased service delivery efficiency (eg, increased treatment availability and decreased waiting times). However, these factors were not widely recorded across the studies. Telehealth was demonstrated to be cost-effective in the few studies that considered cost-effectiveness. Study quality varied, as many studies had small sample sizes and inadequate control groups. Of the 42 studies, only 11 (26%) were randomized controlled trials, 12 (29%) were case studies or case series, 6 (14%) were qualitative studies, and 5 (12%) were noncomparative trials.

**Conclusions:**

Telehealth has the potential to increase treatment availability, decrease diagnosis waiting times, and aid in NDD monitoring. Further research with more robust and adequately powered study designs that consider cost-effectiveness and increased efficiency is needed. This systematic review highlights the extent of telehealth technology use prior to the COVID-19 pandemic and the movement for investing in remote access to treatments.

**Trial Registration:**

PROSPERO International Prospective Register of Systematic Reviews CRD42018091156; https://www.crd.york.ac.uk/prospero/display_record.php?ID=CRD42018091156

## Introduction

### Background

Neurodevelopmental disorders (NDDs) are lifelong disorders that typically develop during the early stages of child development and have a high frequency of co-occurrence [1,2]. In this systematic review, NDDs are defined in accordance with the Diagnostic and Statistical Manual of Mental Disorders, Fifth Edition criteria [3], and include autism spectrum disorder (ASD), attention deficit hyperactivity disorder (ADHD), intellectual disability, communication disorders, specific learning disorder, motor disorders, stereotypical movement disorder, and tic disorders. Young people with NDDs have been identified as particularly vulnerable to the mental health impacts of COVID-19, due to changes in support and routine and increased isolation and loneliness [4,5].

Prior to the COVID-19 pandemic, telehealth interventions were attracting interest as effective options for improving mental health provision in overstretched health services. The COVID-19 pandemic has increased the demand for effective mental health support, and the growing need to offer easy-to-access remote service availability [4,6,7] has substantially increased telehealth use [8]. It is therefore essential that we not only identify which existing telehealth technologies show the greatest efficacy for use with individuals with NDDs, but also capture the state of the existing evidence base in order to evaluate the inevitable growth of this field.

### Prior Work

There is no universally agreed upon definition for telehealth [9]. In this systematic review, we use the term “telehealth” to encompass telemedicine, telemental health, and telepsychiatry.

In a systematic review of the use of telehealth services for communication disorders, Molini-Avejonas and colleagues [10] found that over 85% (88/103) of telehealth studies reported the advantages that telehealth has over nontelehealth approaches. For example, Molini-Avejonas and colleagues [10] reported that telehealth is typically viewed favorably by users and health care practitioners, as telehealth helps to reduce geographical barriers and possibly save time during consultations and travel. However, barriers to telehealth implementation have been identified. These barriers relate to training, technology issues, and acceptance by both health care practitioners and patients [10]. Indeed, a study that explored the views of health care practitioners (ie, neurologists) toward digital devices in clinical practice found that while the majority (95%) of the 405 participants used computers regularly at work, less than half (43.5%) used a tablet [11]. This suggests that one of the barriers to the uptake of technology may be acceptance from health care professionals.

Sutherland and colleagues [12] have also updated a systematic review [13] of telehealth literature on participants with ASD. During 2010-2016, 14 studies with a total of 284 ASD participants assessed telehealth services, including assessments, interventions, functional behavioral analyses, and language therapy. These studies included a variety of controls, including comparisons between telehealth and face-to-face sessions (6/14, 43%), online learning with and without telehealth sessions (6/14, 43%), and telehealth services that provided no intervention and those that provided treatment as usual (2/14, 14%). Although these studies varied in quality, telehealth services were comparable to face-to-face services and better than control/comparison groups in experimental studies. Another systematic review found that telehealth systems have been used to deliver education to parents and support the diagnosis and treatment of ASD [14].

In terms of ADHD, only 1 systematic review has focused on the use of telehealth. This review found 11 articles, which all reported data from 3 trials that were conducted in 2007-2017 [15]. The majority (10/11, 91%) of studies used a sample of children. Telehealth was viewed favorably, as it was well accepted by health care professionals and users and shown to provide improved outcomes, such as reduced symptomology and improved functioning. However, the authors concluded that further research was necessary to assess the usefulness of telehealth in health care delivery [15]. This review highlighted a lack of research on using telehealth to replace usual treatment rather than augment usual treatment, and a lack of studies that consider the assessment, diagnosis, and treatment of adults with ADHD.

Although condition-specific systematic reviews have been conducted, no single review has assessed the use of telehealth across people with different NDDs. This is important, given the prevalence of NDD comorbidities. Many previous reviews have also been limited to trials. Although trials are important, user feedback, economic impact, and readiness for clinical adoption are important for rapidly developing policies for implementing telehealth services after the COVID-19 pandemic.

### The Goal of This Study

The aim of this systematic review was to highlight how telehealth has been used, prior to the COVID-19 pandemic, with clinical samples within the neurodevelopmental field, including patients with NDD, their families, and health care professionals. In light of the post-COVID-19 pandemic call for implementing the rapid adoption of telehealth into clinical practice [16], this systematic review focused on studies that reported on the clinical/service effectiveness, economic impact, and user impact (ie, feasibility/acceptability) of telehealth to aid in assessment, diagnosis, monitoring, and treatment. This review serves to identify potentially effective telehealth technologies for use with patients with NDDs and document the evidence base prior to the anticipated rapid expansion of telehealth in the neurodevelopmental field.

## Methods

### Study Design

This systematic review was part of a larger review [17], which assessed all technology that has been used for NDDs*.* The protocol for our main review was registered with PROSPERO (CRD42018091156). Given the vast number of obtained papers that related to telehealth, it was most appropriate to present these in a stand-alone article. The literature search was undertaken in accordance with the recommended principles in the Preferred Reporting Items for Systematic Reviews and Meta-Analyses (PRISMA) guidelines [18].

### Search Strategy

A systematic search of literature was undertaken by an information specialist (EY) using the following databases: Medline, Web of Science, Scopus, CINAHL Plus, EMBASE, and PsycInfo. Searches were also performed in the Cochrane Library, Journal of Medical Internet Research, Institute of Electrical and Electronics Engineers, and Association for Computing Machinery Digital Library databases. The search included all terms that related to NDDs and telehealth, including controlled vocabulary headings such as “Intellectual Disability,” “Mentally Disabled Persons,” “Learning disorders,” “Developmental Disabilities,” “Neurodevelopmental Disorders,” and “Telemedicine.” Keywords and synonyms that related to all NDDs, including “ASD,” “ADHD,” “Tic Disorders,” “Communication and Language Disorders,” “Learning Disorders,” and “Learning Disabilities,” were also used for the search. Terms that related to telehealth included keywords, such as “tele care,” “tele coaching,” “telecomm,” “teleconference,” “teleconsultation,” “telehealth,” and “telemanagement,” as well as terms that related to teletherapy, telepractice, and eHealth. As this study was part of a wider search of all technologies, additional terms that related to various technologies, such as mobile apps, video games, virtual reality, and robotics, were also included. However, the results of the search for these terms are presented in another study [17]. A copy of the Medline search strategy is included in Multimedia Appendix 1. Endnote software (Clarivate) and Microsoft Excel were used to manage the data. The initial search was restricted to published, peer-reviewed, academic papers written in English, and was conducted in March/April 2018 and recently updated in July 2020 to cover the period of January 2014 to December 2019. The World Health Organization has acknowledged December 2019 as the month that the first case of COVID-19 was officially recorded [19].

PICOS (population, intervention, comparison, outcome, study design) guidelines were used to define the inclusion criteria. With regard to population, we included studies that involved people with NDDs or parents, carers, or health care professionals who worked with people with NDDs. With regard to intervention, we included studies that clinically used telehealth equipment in the assessment, diagnosis, monitoring, or treatment of NDDs. No restrictions on comparisons were put in place for literature. With regard to outcomes, included studies were to have at least 1 outcome of interest from clinical effectiveness, economic impact, and user impact. Based on the National Institute of Clinical Excellence glossary, the following terms were referred to in the search: (1) “clinical effectiveness,” which refers to how beneficial telehealth was in terms of assessment, monitoring, or treatment compared to usual care, a control group, or another type of care; (2) “economic impact,” which refers to the evaluation of service delivery efficiencies (eg, whether an intervention reduces clinician time), as well as any economic evaluation (eg, cost-effectiveness or costs and benefits evaluations) of telehealth; and (3) “user impact,” which refers to the feasibility of using telehealth in terms of technical feasibility (ie, how simple or difficult it was to use telehealth services) and the administrative infrastructure (ie, how the technology fits within an organization). Usability impact also covered design factors that affect the user experience and users’ acceptability of the technology (ie, users’ willingness to attend and engage with the technology). With regard to study types, we excluded systematic reviews and meta-analyses.

Studies on telehealth were restricted to those that used synchronous (ie, real-time) remote clinical care in relation to the diagnosis, monitoring, or treatment of an NDD. Although studies that involved both audio and video communication were included, studies that provided care via only a telephone were excluded. Studies were also excluded if they used asynchronous (ie, nonreal-time) data, including email communications between patients and health care practitioners, physiological data (eg, electroencephalogram data) that were remotely interpreted, and data regarding telehealth services that were delivered solely in educational/employment settings, such as schools or vocational training centers. In addition, studies were excluded if they did not involve an NDD clinical sample or if they focused on lifestyle interventions (eg, obesity management rather than NDD treatment).

### Data Extraction and Quality Assessment

Titles and abstracts were reviewed for initial screening, and excluded papers were further independently screened. Two authors (AZV and CLH) independently reviewed full texts and extracted data by using an Excel database. Extracted data included authors and the year of publication; brief summaries of the study design, including the type of telehealth used and study methods; how the technology was used (ie, assessment, treatment, or monitoring); and information on participant samples, including the number of participants in a sample, health condition, gender, population type (ie, parent, clinician, or children/young people populations), and age (ie, if children were studied). The relevant outcomes that related to the authors’ views on clinical effectiveness, user impact, economic impact, and readiness for clinic adoption were also noted. Results were synthesized in tabulated form (Multimedia Appendix 2).

A quality review of the research was also conducted. Papers were appraised by 3 authors (CLH, SSH, and BJB) based on the Oxford Centre for Evidence-Based Medicine Levels of Evidence. Each paper was rated with a score of 1-5; randomized controlled trials (RCTs) were typically ranked high (score=1) and qualitative papers/judgments were typically ranked low (score=5). Throughout the paper, this score is referred to as a quality rating (QR) [20]. Disagreements were resolved through discussion.

## Results

**Figure 1:**
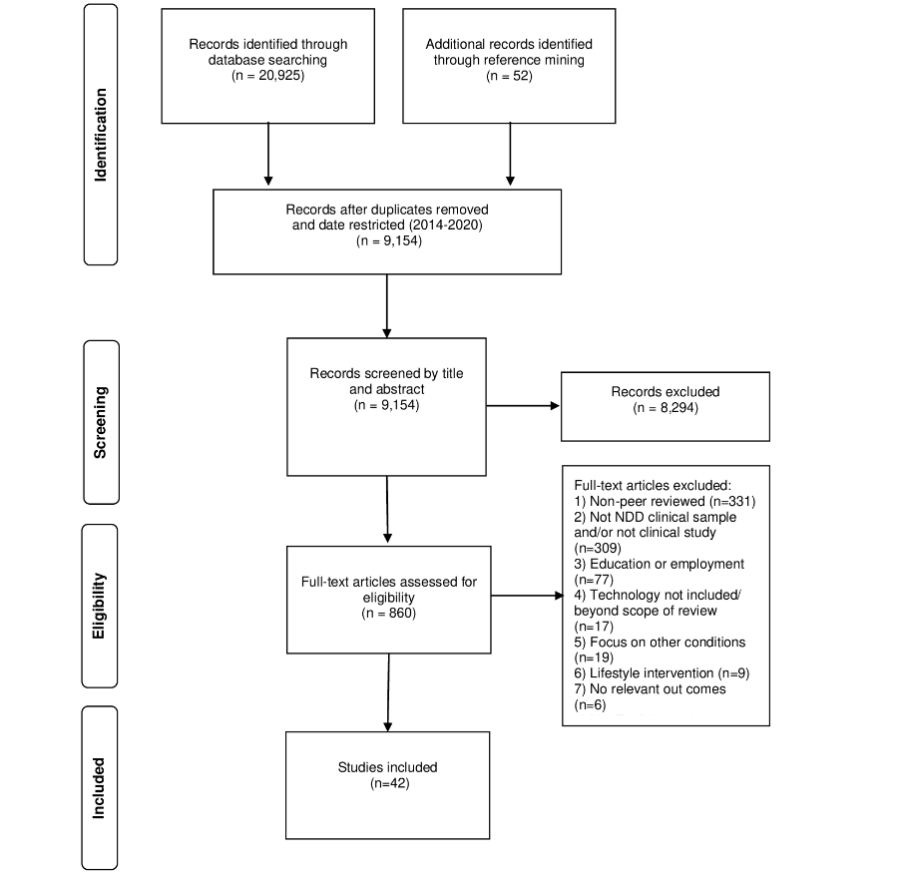
PRISMA (Preferred Reporting Items for Systematic Reviews and Meta-Analyses) 2009 flow diagram depicting the study selection process.

The process of identifying and selecting studies is outlined in a flow diagram (Figure 1), and a summary of the included papers is presented in Multimedia Appendix 2.

### Study Characteristics

A total of 42 studies met the inclusion criteria. The greatest number of studies were conducted on ASD (22/42, 52%) and ADHD (8/42, 19%). Studies on communication disorders (7/42, 17%), and tic disorders (2/42, 5%) were the least represented. Additionally, 3 (7%) studies used a sample of participants with ADHD, ASD, or both. Of the 42 papers, 23 (55%) reported a wide range of additional diagnoses, such as another coexisting NDD (10/23, 43%), oppositional defiant disorder (7/23, 30%), and anxiety (4/23, 17%), and 19 (45%) studies did not report any comorbidities. Most studies (29/42, 69%) reported data from children’s parents/carers. Of these 29 studies, 22 (76%) included children aged <7 years, 3 (10%) included adult telehealth service users, and 7 (30%) documented the perspective of health care professionals. Approximately half (20/42, 48%) of all papers reported on data from a male or predominantly male sample. However, in parent/carer studies, the primary caregiver was more likely to be female. Most studies were conducted in the United States (27/42, 64%), Australasia (7/42, 17%) and Europe (6/42, 14%). Studies typically focused on treatment (33/42, 79%), rather than monitoring (5/42, 12%) or assessment (4/42, 10%).

### Outcomes of interest

#### Assessment

##### Summary of Assessment Papers

We found 4 papers that focused on the assessment of NDDs. Of these 4 papers, 3 (75%) used telehealth to remotely diagnose ASD [21-23] and 1 (25%) assessed the objective measurement of hyperactivity in patients diagnosed with ADHD [24]. All ASD studies involved parents and children under 6 years of age. The ADHD study involved children and young people aged 6-16 years. All studies had ≤65 participants (range 17-65; Multimedia Appendix 2). Of the 4 papers, 3 (75%) had a QR of 2 [22] or 3 [23,24], and 1 (25%) [21] had the lowest QR of 5.

##### Clinical Effectiveness

The Wehrmann and Müller [24] pilot non-RCT used webcam footage to create a video-activity score to measure physical activity as an objective assessment of hyperactivity in children with suspected ADHD. The video-activity score did not show criterion validity with clinicians’ or parents’ hyperactivity ratings.

The findings from the ASD studies were more favorable. Reese and colleagues [22], who reported preliminary RCT findings on which families were assigned to in-clinic or telehealth evaluations, found that families could be coached to complete ASD assessment activities with young children via videoconferencing and clinicians could make accurate diagnoses remotely. Similarly, Juarez et al [21] reported on 2 studies, of which 1 compared a telediagnosis to a face-to-face assessment. This study demonstrated that, compared to gold-standard tools, remote ASD diagnostic consultations resulted in clinicians correctly diagnosing 78.9% (15/19) of children. No children were inaccurately diagnosed with ASD. Stainbrook and colleagues [23] investigated referrals before and after the introduction of a telehealth service. They found that implementing a diagnostic consultation service for ASD, in partnership with an early intervention service, increased referrals for diagnostic evaluation and the likelihood of families attending appointments. Following referral, 56 (89%) of the 63 families chose to receive further appointments via telehealth services rather than face-to-face services, and families with complex problems were the most likely to access clinic services.

##### User Impact, Feasibility, and Acceptability

In a second qualitative feasibility study, Juarez and colleagues [21] reported positive user feedback from both health care professionals and families. Families from rural areas reported geographical and time barriers to accessing traditional health care. These barriers were reduced with remote diagnoses, leading to high levels of satisfaction. Stainbrook and colleagues [23] found that families were more likely to attend telehealth appointments. Following referral, 56 (89%) of the 63 families chose to receive appointments via telehealth services rather than face-to-face services, and families with complex problems were the most likely to access clinic services.

##### Service Delivery Efficiencies and Economic Impact

Stainbrook and colleagues [23] were the only authors to document service delivery efficiencies. They reported that implementing a telehealth service reduced the time to diagnosis by 11-12 months.

##### Readiness for Clinic Adoption

Despite the effectiveness and positive user impact of telehealth in the assessment of ASD, the studies all had a small sample size. Of the 3 ASD papers, 1 (33%) did not report on suitability for implementation [23] and 2 (67%) stated that further research is necessary [21,22]. The ADHD assessment paper [24] reported negative findings and concluded that telehealth assessments for ADHD were not suitable for implementation. As such, prior to the COVID-19 pandemic, there were no telehealth technologies that were reported to be efficacious in assessing NDDs and suitable for immediate adoption in practice.

#### Monitoring

##### Summary of Monitoring Papers

We found 5 articles that reported on a sample of children with ADHD and their families. All papers were based on the CATTS (Children’s ADHD Telemental Health Treatment Study) [25]. We found an RCT that assessed the effectiveness of a telehealth service for children with ADHD, which included pharmacological treatment monitoring and caregiver behavior training/psychoeducation. The RCT study compared families who received augmented treatment as usual, which involved only 1 telehealth consultation, to families who received 6 telehealth sessions, which were conducted approximately 1 month apart. The papers were generally highly rated (QR=2), and the main study was an RCT. Secondary papers looked at caregiver outcomes [26,27] and health care professionals’ decisions on medication changes [28]. The remaining paper received a low QR (QR=5) because of the qualitative nature of the report, which focused on caregiver satisfaction and engagement, and health care professional fidelity [29]. Although not all studies were directly related to monitoring, they were collated together to allow the reader to understand that data were from multiple articles that related to the same trial. We found 4 studies that were based on the main trial’s dataset, which included 223 families of children with ADHD aged 5-12 years and their carers [25]. The remaining study [27] involved a subsample of 37 participants.

##### Clinical Effectiveness

Overall, both methods of telehealth delivery resulted in reductions in ADHD and oppositional defiant disorder behaviors and improvements in role performance and impairment, with the telehealth model generally resulting in better outcomes [25] and better parental mental health [26] than face-to-face models. In a feasibility trial of a subsample of families, Tse and colleagues [27] assessed the outcomes from baseline to 25 weeks and found similar outcomes for child ADHD behaviors. However, they also found that parents who used telehealth services had considerably less improvement in caregiver strain and empowerment than those who received face-to-face training.

##### User Impact, Feasibility, and Acceptability

High levels of engagement and satisfaction were reported by parents [27,29]. Rockhill et al [28] reported that fidelity was not impacted by telehealth delivery. The authors argued that telehealth provides added value in terms of increasing treat-to-target goals and offering support to health care professionals.

##### Service Delivery Efficiencies and Economic Impact

Service delivery efficiencies and economic impact were not reported in any monitoring papers.

##### Readiness for Clinic Adoption

Myers and colleagues [25] provided clinical guidance regarding the use of telehealth in treating children with ADHD, and the results from the RCT were promising. Further research is recommended in the development of the CATTS trial, including research that involves the greater use of teachers in interventions and objective school outcome measures, such as the completion of homework and behavioral observations, to further validate the tool. Tse and colleagues [27] concluded that telehealth delivery was promising in terms of readiness for clinic adoption, but telehealth for caregivers’ distress needed further study, including the investigation of the best delivery modality. Future research on the cost benefits of telehealth models of care for ADHD was also recommended. These findings indicated promise in the implementation of technologies for monitoring ADHD.

#### Treatment

We found 33 papers that reported on the use of telehealth technologies to treat NDDs. The majority of the papers focused on ASD (18/33, 55%). Other reported conditions were ADHD with or without ASD (5/33, 15%), communication disorders (7/33, 21%), tic disorders (2/33, 6%) and learning disabilities (1/33, 3%). Due to the volume of treatment papers, each condition will be considered in turn.

#### ASD

##### Summary of ASD Treatment Papers

In terms of ASD, 1 paper presented a case report of a 16-year-old male with Asperger syndrome, social isolation, and depression. Clarke [30] reported that communicating via telehealth allowed a clinician to develop a relationship with a young person who was later able to attend a clinic in person and reconnect with his family. The remaining papers (17/18, 94%) focused on some aspect of parent training. Of these 17 papers, 6 (35%) reported on providing telehealth-delivered functional analysis and communication training to parents [31-36], and 1 (6%) reported on using telehealth-delivered functional analysis to train a health care professional [37]. These studies mainly consisted of case studies or case series (6/7, 86%) that used a multiple baseline experimental design and had a QR of 3 [35] or 4 [31,32,34,36,37]. Another paper (1/7, 14%) reexamined 2 nonresponding participants’ data from an RCT (QR=4) [33].

We found 4 studies on 4 programs that incorporated self-directed online learning with remote therapy, support, or coaching [38-41]. These included studies on ImPACT Online Communication Training [38,39], which evaluated the feasibility (QR=4) and clinical efficacy (QR=3) of the ImPACT Online program in addressing social communication development, and a noncomparative feasibility study (QR=4) on OASIS ABA (Online and Applied System for Intervention Skills Applied Behavior Analysis)–based parent training [40]. Another program, which involved reciprocal imitation training, was used in a single-subject multiple-baseline design study (QR=4) [41].

In a noncomparative trial that gathered data from before and after intervention (QR=4), Little and colleagues [42] studied occupational-based coaching via telehealth for increasing positive interactions and everyday routines. This included an evaluation of acceptability/cost [43] and a linked qualitative (QR=5) appraisal of parents’ perceptions [44]. The remaining programs were the Sunny Starts parent training program for increasing sociocommunicative behavior, which was used in a case series with multiple baseline experimental data (QR=4) [45]; the RUBI-PT (Research Unit on Behavioral Interventions-Parent Training) program, which was developed by the Research Unit on Behavioral Interventions Autism Network and targeted behaviors such as aggression and tantrums in children with ASD; benchmarking, which was used in a trial that compared the data of new services to data from previously published clinical trials (QR= 4) to evaluate effectiveness (eg, reduction in disruptive behavior), feasibility, and acceptability [46]; and parent coaching with a focus on educating parents about effective approaches for children with ASD (eg, social narratives and visual schedules), which was used in a qualitative paper (QR=5) [47].

##### Clinical Effectiveness

The majority of the ASD papers reported that treatment was clinically effective in improving caregiver knowledge, caregiver competence, and child participation (6/18, 33%) [38-42,45], increasing communication responses (2/18, 11%) [34,45], and reducing problem behaviors (5/18, 28%) [31,32,35-37]. We found 1 (5%) paper [33] that discussed 2 young children with ASD who underwent functional communication training, but this was unsuccessful in reducing problem behaviors. The authors suggested that although not all patients can be treated via telehealth, if sessions are recorded, watching the recordings can lead to the identification of the reason why treatment was not successful. Ingersoll and colleagues [39] noted that both online self-directed training and therapist-assisted, parent-mediated telehealth intervention led to improvements in fidelity, self-efficacy, stress, and parents’ perceptions of their child, and that families who received therapist coaching and support gained improved social skills.

##### User Impact, Feasibility, and Acceptability

High levels of engagement and satisfaction were reported by parents [40,43,46]. However, difficulties surrounding failing technology and incomplete personal interaction were also documented [44,47]. Ingersoll and colleagues [38] found that parental engagement and satisfaction were similar for both self-directed and therapist-assisted methods of telehealth delivery. However, having a therapist increased engagement and led to higher rates of telehealth service completion. Parents often engaged with the program (ie, without therapist support) outside of traditional working hours, which allowed for greater flexibility than in face-to-face coaching.

In a qualitative study, Ashburner et al [47] explored the perceived advantages and disadvantages of a follow-up early intervention service that was delivered via remote technology, by comparing the service to previous face-to-face services. Content analysis showed that parents, service providers, and the ASD specialist perceived remote technologies to be helpful in upskilling parents/service providers and enabling families to access support from home. However, all study participants agreed that remote technology should be used to augment, rather than replace, face-to-face contact, which is similar to the findings reported by Little et al [43].

The use of telehealth for training health care professionals was also shown to be a promising way of providing support to practitioners in the field, which led to the greater implementation of target strategies [37].

##### Service Delivery Efficiencies and Economic Impact

Suess and colleagues [32] reported on telehealth service delivery efficiencies and argued that in some cases, brief, efficient telehealth appointments bypasses the need for further in-clinic support and allows for quicker treatment initiation. Several authors [40,41,47] suggested that telehealth has the potential to increase access to ASD services (ie, particularly in remote areas) and reduce costs, time, and travel. Lingren et al [35] compared the costs of therapy for caregivers of children with ASD between different telehealth models, including in-home telehealth, regional clinic telehealth, and in-home, face-to-face telehealth models. The costs were lowest for the in-home telehealth model, but the in-home and in-home, face-to-face telehealth models were substantially less costly than the costs for face-to-face in-home therapy. Similarly, in a study that involved a 12-week telehealth intervention for families with a child with ASD, the authors reported that the costs for both outpatient and in-home care models were approximately 2.6 times more expensive than the costs for telehealth models [43].

#### ADHD

##### Summary of ADHD Treatment Papers

We found 5 treatment papers from the ADHD sample that included patients with ADHD and patients with ASD. The highest quality paper (ie, a small RCT with a QR of 2) compared patients who underwent internet-based cognitive behavioral therapy based on the InFocus program (ie, with/without therapist support) to those in the waitlist control [48]. The other papers were of much lower in quality. We found 1 experimental pilot study (QR=5) that used a nonrandomized pre-post intervention study design to assess the feasibility and acceptability of a parenting group training program delivered via telehealth [49]. Additionally, Sehlin and colleagues [50] provided qualitative data (QR=5) for a study that involved a face-to-face meeting that was followed by 8 weeks of internet-based chat sessions for providing coaching and support. Another qualitative paper (QR=5) conducted implementation interviews with health care professionals after providing coaching and support at 3 trial sites in Sweden [51]. The final paper used a multiple descriptive case design (QR=4) to assess caregiver perspectives in a sample of 10 caregivers of young people with ADHD or ASD who took part in an internet-based intervention [52].

##### Clinical Effectiveness

In general, clinical effectiveness was unclear or not reported (3/5, 60%) [50-52], and all studies were limited by small sample sizes (range 7-45). We found 2 group therapy telehealth programs that showed great promise. A study [48] found that an internet-based cognitive behavioral therapy telehealth treatment program, which included weekly online group therapy sessions for adults with ADHD, was no more clinically effective than unsupported self-help alone. However, people in both programs faired better than those in the waiting list controls. The second group therapy program showed a trend of improvement in child ADHD symptoms following a group parenting intervention, but the program was not adequately powered [49].

##### User Impact, Feasibility, and Acceptability

Sehlin et al [50] found that although remote coaching was perceived favorably by participants, difficulties surrounded failing technology and incomplete personal interaction were reported. Shah and colleagues [49] also reported that clinicians experienced difficulties with internet connections and found it hard to read body language and expressions, as faces were sometimes out of focus during video appointments. They also reported that patients experienced disturbances from other family members, and that the inability to role play during telehealth appointments was problematic. However, parents were at ease and relaxed during telehealth appointments.

Gillberg and Wentz [51] assessed professionals’ perceptions on internet-based support and coaching and the barriers and facilitators to implementation. Facilitators of positive perceptions included improved access, equality distribution, and the delivery/quality of health care services. Reported barriers included the design of the intervention, technical issues, attitudes of staff, organizational culture and structure, and work division and resource allocation.

##### Service Delivery Efficiencies and Economic Impact

Most studies (4/5, 80%) did not report the economic impact. However, cost-savings in terms of time and travel were noted in 1 (20%) study [49].

#### Other NNDs (ie, Communication Disorders, Tic Disorders, and Learning Disabilities)

##### Summary of Other Treatment Papers

Treatment programs for communication disorders (eg, stuttering) were evaluated in 7 papers. A noncomparative trial (QR=4) investigated the Camperdown Program, which was used to reduce stuttering in adolescents [53]. The remaining papers assessed the Lidcombe program for preschoolers. With regard to the Lidcombe program, we found 1 RCT (QR=2) that compared telehealth care delivery to in-clinic, face-to-face care delivery [54], and 1 study (QR=2) that involved a quantitative evaluation of parent satisfaction ratings [55]. We found 1 paper (QR=4) that involved a noncomparative trial that assessed reductions in stuttering severity and frequency, as well as satisfaction with telehealth delivery [56]. The remaining papers all had the lowest quality rating (QR=5). We found 2 papers that discussed clinical insights from health care professionals who were involved in telehealth treatment delivery for patients with stuttering [57,58]. We also found a descriptive-analytic study of satisfaction with telehealth treatment for stuttering [59]. Furthermore, we found 2 papers on a pilot open-case series (QR=4) [60] and an RCT that used a waiting list control for the assessment and treatment of chronic tic disorders (QR=2) [61]. The final paper (QR=5) provided an account of a telehealth service that was delivered at a large-scale regional service level [62].

##### Clinical Effectiveness

In terms of the Lidcombe program, the Phase I [56] trial demonstrated the efficacy of remotely delivering the program to families with a preschool child who stutters. However, the results of a main parallel, open-plan, noninferiority RCT trial [54] showed that it was not clear whether webcam treatment was noninferior to standard treatment in the short term. Carey and colleagues [53] conducted a Phase II clinical trial that examined adolescents’ responsiveness to the webcam-delivered Camperdown program, and found that adolescents experienced substantially reduced stuttering in terms of both frequency and severity, although relapse was a problem.

##### User Impact, Feasibility, and Acceptability

High levels of engagement and satisfaction were reported by parents/carers [53,60,61]. The use of telehealth to train health care professionals was shown to be a promising way of providing support to practitioners in the field [58]. Jahromi and Ahmadian [59] explored satisfaction in telespeech therapy among 30 Iranian patients aged ≥14 years. The authors reported that satisfaction with the therapy was high, but the low internet speed in the country was a major challenge for half the participants, as they could not maintain eye contact with the therapist due to the distorted image transmission. Similarly, another study reported that difficulties arose with regard to completing certain aspects of treatment due to limited web camera viewing ranges and audio/visual difficulties [61].

The feasibility of delivering both the Lidcombe and Camperdown programs via telehealth methods was documented, and parents were generally satisfied [53,54,56]. However, Bridgman and colleagues [57] highlighted that individual adjustments were required to tailor the treatment process to families’ needs in order to maximize outcomes. Ferdinands and Bridgman [55] examined parent satisfaction and stuttering severity at baseline and during the 9-month/18-month follow up, and found that increased parental satisfaction was generally, but not always, linked with the severity of stuttering. This demonstrates the need to provide treatment at the family level when monitoring children with communication disorders. There was no considerable difference in parent satisfaction between clinic and telehealth care delivery.

Ricketts et al [60,61] conducted pilot studies that explored the feasibility of assessing tic severity over voice over internet protocol (VoIP), which allows users to make and receive calls via an internet connection. They compared the feasibility, acceptability, and efficacy of VoIP-delivered therapy for tic disorders to those of a waitlist control. They found a decrease in tic severity that was similar to the decrease identified in the original Cognitive Behavioral Intervention for Tics trial [63] and greater than that of the waitlist control [61].

##### Service Delivery Efficiencies and Economic Impact

Merrill et al [62] provided an overview of Ohio’s Telepsychiatry Project for Intellectual Disability, which provides specialized mental health services to rural communities. This paper documented telehealth from a service delivery perspective. Although no specific figures were given, the report indicated that the service improved access to care, reduced emergency department visits/hospitalizations, and resulted in cost savings, including reduced travel expenses, medical expenses, and support costs. Similar cost savings were reported in other studies [54,59].

##### Readiness for Clinic Adoption

Of the 33 treatment papers, 5 (15%) deemed telehealth to be suitable for clinic adoption, either as an adjunct to current practices or on its own [30,47,50,59,62]. Furthermore, 24 (73%) papers noted that telehealth required further research before being implemented into clinical practice. The remaining papers were unclear/did not report on readiness for clinic adoption. Prior to the COVID-19 pandemic, the delivery of interventions via telehealth for parents of children with ASD, young people and adults who stutter, and adults with intellectual disability were thought to be suitable for clinic adoption. For young people who struggle with attending appointments, therapy conducted via VoIP was recommended. In addition, coaching and support via a chat program was recommended as an adjunct to usual treatment for young people and adults with ADHD/ASD.

## Discussion

### Principal Results

The purpose of this systematic review was to examine the evidence base for the clinical use of technology within the neurodevelopmental field prior to the COVID-19 pandemic, to identify possible telehealth technologies that can be considered for wide-spread implementation and document the current state of the evidence base prior to the anticipated rapid development in this field.

#### Assessment

Telehealth has been used to assess small samples of people with ASD, and telehealth shows promise for clinical adoption. In terms of economic impact, there are potential cost savings and service efficiencies, but the evidence base is limited. The ADHD assessment tool is not clinically effective, and there has been no evidence for the assessment of other NDDs at present.

#### Monitoring

As identified in a previous review by Spencer and colleagues [15], all studies that used telehealth for monitoring were for monitoring ADHD, and all studies were from the same trial (ie, the CATTS [25]), which had promising results for acceptability and effectiveness. Telehealth monitoring seems to be an approach that should be considered for clinic adoption.

#### Treatment

Telehealth has been used to treat a range of NDDs. However, the majority of conditions fall under ASD, and treatment has mainly focused on parent training interventions. These interventions have shown some evidence of clinical efficacy, such as improving caregiver knowledge, competence, and child participation, and reducing problem behaviors. Even when telehealth is not clinically effective, the recording of sessions can help health care professionals identify why the treatment did not work [33]. Treatments for communication disorders have also focused on parent intervention programs, which have shown some evidence of clinical efficacy and no difference in parent satisfaction between remote delivery and face-to-face delivery. Despite the fact that previous literature has suggested that the evidence for using telehealth to manage communication disorders is substantial [10], our review did not reveal a large number of papers that involved communication disorders, as more papers focused on ASD. Furthermore, our findings on effectiveness were mixed; the lack of an adequate control group was a limiting factor in several studies [53].

We found little evidence for the delivery of parenting interventions for ADHD. However, it is possible that the search terms used in this review limited access to such papers. Telehealth services for young people and adult service users tended to focus on the remote delivery of coaching, support, and therapy. The 1 case study of a young person with ASD who received online therapy had a promising outcome. People with ASD may particularly benefit from using technology to overcome communication difficulties, as this involves fewer social pressures than face-to-face therapy [64].

Cognitive-behavioral strategies have been used for both ADHD and tic disorders, and mixed clinical efficacies have been reported. There is a larger body of evidence for using behavioral and cognitive-behavioral treatments for tic disorders than evidence for using such treatments for ADHD, but further research is necessary for both disorders. There was limited evidence for using telehealth as a means of providing training to health care professionals. However, barriers to this approach, including the design of the intervention, technical issues, attitudes of staff, organizational culture and structure, and work division and resource allocation, were widely reported.

In summary, there is a much larger body of evidence for the efficacy of providing remotely delivered interventions to parents and children than evidence for providing such interventions to young people and adult service users. There is also a small body of evidence for using telehealth to train health care professionals. Generally, the user impact for all participants was positive. There was very little research on economic impact. Overall, the evidence base is of variable quality.

### Key Implementation Issues

This systematic review highlighted key implementation issues for using telehealth services. The number of telehealth technologies that are ready to be implemented in practice is limited, as most studies stated that further research is necessary before such technologies are acceptable for clinical adoption. Service providers should consider both service users’ opinions on such technology and the evidence base when choosing whether to implement telehealth technology into clinical practice. If families view telehealth technology as an adjunct to usual treatment, cost savings may not be achieved.

The telehealth delivery of treatments may have benefits. In some studies, allowing users to access treatment at convenient times and providing personalized treatment led to greater treatment engagement and completion. Families were more at ease and relaxed when participating in telehealth treatment. The need to personalize treatment to individuals and families was apparent across several studies. This is particularly important, as disruptions by other family members can occur. There is limited evidence for service delivery efficiencies. Implementation difficulties included failing technology, audio and visual problems, and difficulties in making eye contact. These were particularly problematic in countries with low internet speeds. In line with previous reviews, several studies have reported that health care professionals found reading body language and facial expressions difficult due to distorted images [10,65].

### Directions for Future Research

This systematic review reveals that there is a lack of research that assesses the use of telehealth in aiding the diagnosis of a wide range of NDDs, and that the current focus is on autism. In general, cost-effectiveness and possible service efficiencies are underinvestigated, but they are an important consideration for real-world implementation. Future research should focus on developing guidelines and blueprints for how to best integrate telehealth care into clinical practice [66].

### Limitations

The limitations of this study must be taken into account when interpreting the findings. As the search yielded a much greater number of papers than anticipated, the search was limited to the previous 5 years. This was a deviation from the initial protocol. However, it can be argued that this method allows for a more effective analysis of current technology and precludes the inclusion of outdated technology. Furthermore, limiting the search to published academic papers may have exacerbated the risk of bias, as authors were not contacted for unpublished work due to the volume of published papers obtained. This is a limitation of our study, and further reviews should explore unpublished data, especially data from conference papers, as these provided a vast amount of possibly relevant data. However, conference papers were excluded from this systematic review due to time constraints.

The majority of studies were conducted in high-income countries, thereby limiting the generalizability of our findings. It is likely that there would be intercountry variations in barriers to implementing new technology into existing health care systems. Although these barriers are typically considered outside the remit of standard reporting for trials, an understanding of these barriers is important if these technologies are to be routinely implemented.

The majority of data were of mid- to low-quality, and our findings should be interpreted with caution. This was generally because of small sample sizes and the high number of qualitative/reflexive study designs. However, RCTs are time-consuming and do not always lend themselves to real-world evaluations.

### Conclusions

Our literature search highlighted that, prior to the COVID-19 pandemic, there was promising evidence for the use of telehealth in clinical practice, in relation to NDDs. Telehealth technologies were more frequently used to support the treatment and monitoring of NDDs; there was less evidence for their use in supporting the assessment of NDDs. The main focus of telehealth in the neurodevelopmental field was on ASD and ADHD, which are two of the most commonly occurring NDDs. There was evidence of good clinical outcomes and cost savings for health care providers. However, further research is required to substantiate this evidence. With the growing need to provide easy access to remotely delivered clinical support for enabling the wide-spread reach of health care and reducing the risk of spreading infectious diseases, it is essential that real-world evaluations for implementation and cost-effectiveness are conducted.

## Appendix

Multimedia Appendix 1 Sample search for 1 database (ie, Medline).

Multimedia Appendix 2 Summary of included studies.

## Abbreviations

ADHD: attention deficit hyperactivity disorder
ASD: autism spectrum disorder
CATTS: Children’s ADHD Telemental Health Treatment Study
NDD: neurodevelopmental disorder
OASIS ABA: Online and Applied System for Intervention Skills Applied Behavior Analysis
PICOS: population, intervention, comparison, outcome, study design
PRISMA: Preferred Reporting Items for Systematic Reviews and Meta-Analyses
QR: quality rating
RCT: randomized controlled trial
RUBI-PT: Research Unit on Behavioral Interventions-Parent Training
VoIP: voice over internet protocol
