# Supplementary material for: Implementation of Telehealth Services to Assess, Monitor, and Treat Neurodevelopmental Disorders: Systematic Review
Source: J Med Internet Res. 2021 Jan 20;23(1):e22619. doi: 10.2196/22619 (PMC7819544; doi:10.2196/22619)
Supplement: Multimedia Appendix 1 [file jmir_v23i1e22619_app1.doc]

1 exp Intellectual Disability/

2 Mentally Disabled Persons/

3 Learning disorders/

4 Developmental Disabilities/

5 exp Neurodevelopmental Disorders/

6 (mental* adj3 (retard* or disab* or defici* or handicap* or impairment* or subnormal* or sub-

normal*)).tw.

7 ((intellectual* or learning*) adj3 (impair* or disab* or disorder* or difficult* or subnormal* or sub-

normal*)).tw.

8 exp autism spectrum disorder/ or exp autistic disorder/

9 exp ASPERGER SYNDROME/ or exp Child Development Disorders, Pervasive/

10 (autistic or autism or asperger*).tw.

11 "pervasive development* disorder*".tw.

12 Rett*.tw.

13 exp Attention Deficit Disorder with Hyperactivity/

14 exp "Attention Deficit and Disruptive Behavior Disorders"/

15 (attenti* adj2 (deficit* or disorder*)).tw.

16 (adhd or addh or "ad hd" or ad??hd).tw.

17 exp Hyperkinesis/

18 ((hyperkin* or "hyper kin*" or hyper-kin*) adj2 (syndrome* or disorder*)).tw.

19 exp Tic Disorders/

20 exp TOURETTE SYNDROME/

21 exp Stereotypic Movement Disorder/

22 ("Stereotyp* movement disorders" or "stammering" or "cluttering").tw.

23 (Tourette* or "tic disorder*").tw.

24 exp Communication Disorders/

25 ("Communication Disorder*" or "Language* Disorder*").tw.

26 ("Speech Sound Disorder*" or "Childhood?Onset Fluency Disorder*" or Stuttering or "Speech

articulation disorder*" or "phonological disorder*").tw.

27 "Global developmental delay".tw.

28 exp Language Development Disorders/

29 exp Specific Learning Disorder/

30 "Neurodevelopmental disorder*".tw.

31 "Developmental disorder*".tw.

32 ("Specific Learning Disorder*" or "Specific reading disorder" or "Disorder of written expression" or

"Mathematics disorder").tw.

33 or/1-32

34 exp TELEMEDICINE/

35 exp INVENTIONS/

36 (eHealth or ehealth* or e-health* or e health* or "electronic adj health" or mhealth* or m-health*

or "mobile health*" or "m health" or ePsych* or e-Psych* or (electronic adj psyc*) or eTherap* or

e-therap* or (electronic adj therap*)).tw.

37 ("telebehavio?ral health" or "tele care" or telecare or "tele coaching" or telecoaching or

telecomm* or tele-comm* or "tele conference*" or teleconference* or "tele consultation" or

teleconsultation or "tele health care" or "tele health*" or telehealth* or tele-health or "tele

management" or telemanagement or "tele med*" or tele-med* or "tele mental health*" or

"telemental health*" or telemetry or tele-monitor* or telemonitor* or telepractice or "tele

practice" or tele-psych* or telepsych* or "tele speech" or telespeech or "tele therap*" or tele-

therap* or teletherap*).tw.

38 ((intervention* or invention* or innovation*) and technolog*).tw.

39 exp Mobile Applications/

40 ((app or apps or application) adj2 (smartphone* or smart-phone or mobile* or phone* or sensor*

or software)).tw.

41 exp Video Games/

42 (gaming or gamification or videogam* or computer gam* or video gam* or electronic gam*).tw.

43 exp Videoconferencing/

44 (videoconferenc* or "video conferenc*" or videoconsultation* or "video consultation*" or "video

technolog*" or "video model*" or Skype* or facetime or webex).tw.

45 wearable electronic devices/ or fitness trackers/

46 (smartwatch* or (wearable adj device*) or wearables or "real-time monitoring device*" or

actigraphy or accelerometer*).tw.

47 exp Virtual Reality/ or exp Virtual Reality Exposure Therapy/

48 ("virtual reality" or "augmented reality").tw.

49 ("interactive multimedia" or "interactive software").tw.

50 ("digital media" or "software program*").tw.

51 ((Internet or digital* or online* or on-line or web* or virtual) adj2 (deliver* or information or

communication* or assisted or e-learning or support)).tw.

52 (ipad adj2 (app or apps or application or intervention*)).tw.

53 (Internet adj2 based).tw.

54 (technolog* adj2 (deliver* or information or communication*)).tw.

55 ("interactive technolog*" or "wearable technolog*" or "mHealth technolog*" or "mobile

technolog*" or "sensor technolog*").tw.

56 *Robotics/

57 (robot or robots or robotics).tw.

58 or/34-57

59 33 and 58

60 limit 59 to (english language and yr="2014 – 2019")
