# Supplementary material for: Implementation of Telehealth Services to Assess, Monitor, and Treat Neurodevelopmental Disorders: Systematic Review
Source: J Med Internet Res. 2021 Jan 20;23(1):e22619. doi: 10.2196/22619 (PMC7819544; doi:10.2196/22619)
Supplement: Multimedia Appendix 2 [file jmir_v23i1e22619_app2.doc]

**Table A1: Summary of included studies - Assessment**

| **Surname of 1st author, year and**  **brief description of study** | **Evaluation of study** | **Condition^a^, N, Gender^b^, Population^c^, Age** | **Bias** |
| --- | --- | --- | --- |
| **Juarez, 2018**  Looked at early identification of ASD through telediagnosis compared to blinded gold standard evaluations (n=20). Telediagnosis confirmed by face-to-face assessment in most cases, but 20% diagnosed face-to-face were not diagnosed remotely. Preliminary feasibility and acceptability in rural area assessed and reported favourably (n=45), but clinicians unable to diagnose 13% of children remotely. | Clinical effectiveness: Unclear  Service efficiency: NR  Economic evaluation: NR  User impact: Yes  Adoption: Needs research | ASD  N=65  Mixed^m^  P/C & CYP  1.5-3y | 5 |
| **Reese, 2015**  Examines validity and utility of video conferencing (VC) to diagnose ASD. Involved an ASD assessment protocol using clinical judgement based on DSM-5 criteria, standardized measures and other available information. Excellent inter-rater agreement between VC and in-clinic diagnosis | Clinical effectiveness: Yes  Service efficiency: NR  Economic evaluation: NR  User impact: NR  Adoption: Needs research | ASD  N=17  Mixed^m^  P/C & CYP  2.5-6y | 2 |
| **Stainbrook, 2018**  Looked at tertiary care referrals and usage following the introduction of diagnostic consultation via telemedicine. Screening Tool for Autism in Toddlers & Young Children (STAT, Stone et al., 2000) was used remotely during a 60-minute appointment. The assessor made an ASD diagnosis, ruled out ASD, or recommended further assessment. Telehealth had a positive impact on referrals and improved attendance. | Clinical effectiveness: Yes  Service efficiency: Yes  Economic evaluation: NR  User impact: Yes  Adoption: NR | ASD  N=63  NR  P/C of CYP  2-3y | 3 |
| **Wehrmann, 2015**  Pilot study assessing an objective measure of hyperactivity using a compressed webcam to provide a measure of physical activity (hyperactivity assessment) via video. A 6-minute cognitive performance task was completed. Compared videoactivity scores with student, clinician, and parent ratings, but found poor association suggesting measure assesses physical activity but not hyperactivity. | Clinical effectiveness: Unclear  Service efficiency: NR  Economic evaluation: NR  User impact: NR  Adoption: No | ADHD  N=39  Mixed^m^  CYP  6-16y | 3 |
|  |  |  |  |

NOTES: **^a^Condition:** ASD=autism spectrum disorders, ADHD=attention-deficit/hyperactivity disorder; **^b^Gender:** Mixed^m^=mixed predominately male; **^c^Population:** Participants are defined as those who participated in the intervention. CYP=children and young people, P/C=parents/ caregivers; **^d^Ratings:** YES/NO (was/was not effective/efficient/ acceptable/suitable for clinical adoption). Unclear= some outcomes were beneficial, some were not, or increased costs were seen in one area but saved in another; NR=not reported

**Table A2: Summary of included studies – Monitoring**

| **Surname of 1st author, year and**  **brief description of study** | **Evaluation of study** | **Condition^a^, N, Gender^b^, Population^c^, Age** | **Bias** |
| --- | --- | --- | --- |
| **McCarty, 2015**  Paper describes the 6-session intervention used in the Children’s ADHD Telemental Health Treatment Study (CATTS; see Myers below). Includes assessment on caregiver satisfaction and engagement, and HCP fidelity and feedback, which were all generally positive with HCPs adhering to protocol over 90% of the time. | Clinical effectiveness: NR  Service efficiency: NR  Economic evaluation: NR  User impact: Yes  Adoption: Needs research | ADHD  N=223  NR  P/C & CYP  5-12y | 5 |
| **Myers, 2015**  The effectiveness of the CATTS is presented. CATTS is a RCT which compares two intervention conditions. The telehealth service delivery model, provided 6-sessions over 22 weeks, including pharmacotherapy (drug titration), delivered via videoconferencing and face-to-face behavior training for caregivers (therapists supervised remotely). The control group received management in primary care augmented with a single telepsychiatric consultation. Found all children improved, although the telehealth delivery model improved significantly more in a number of areas. | Clinical effectiveness: Yes  Service efficiency: NR  Economic evaluation: NR  User impact: NR  Adoption: Needs research | ADHD  N=223  Mixed^m^  P/C & CYP  5-12y | 2 |
| **Rockhill, 2016**  Examined whether HCPs decision making about medication changes was associated with baseline ADHD severity, comorbidity, and treat-to-target goal in CATTS. Reported over 90% fidelity to medication management & greater attainment of treat-to-target goal (46% intervention, 14% control). More medication changes made in CYP with higher baseline ADHD severity and comorbid conditions. | Clinical effectiveness: Yes  Service efficiency: NR  Economic evaluation: NR  User impact: Yes  Adoption: Needs research | ADHD  N=223  Mixed^m^  P/C & CYP  5-12y | 2 |
| **Tse, 2015**  Subsample of families from the intervention arm of CATTS, 12 families received caregiver behaviour training via telehealth, 25 face-to-face. Caregiver outcomes at 25-weeks assessed. Attendance & satisfaction comparable across methods of delivery, but caregiver distress did not improve remotely. Feasibility, acceptability and effectiveness are reported positively but further work on caregiver distress necessary. | Clinical effectiveness: Neutral  Service efficiency: NR  Economic evaluation: NR  User impact: Yes  Adoption: Needs research | ADHD  N=37  Mixed^m^  P/C & CYP  5-12y | 2 |
| **Van der Stoep, 2017**  Outlines caregiver outcomes from the CATTS. Found significant improvements in distress, stress, strain, and family empowerment in the telehealth intervention arm. | Clinical effectiveness: Yes  Service efficiency: NR  Economic evaluation: NR  User impact: NR  Adoption: Needs research | ADHD  N=223  Mixed^F^  P/C & CYP  5-12y | 2 |

NOTES: **^a^Condition:** ASD=autism spectrum disorders, ADHD=attention-deficit/hyperactivity disorder; **^b^Gender:** Mixed^m^=mixed predominately male, Mixed^f^=mixed predominately female; **^c^Population:** Participants are defined as those who participated in the intervention. CYP=children and young people, P/C=parents/ caregivers; **^d^Ratings:** YES/NO (was/was not effective/efficient/ acceptable/suitable for clinical adoption). Neutral = did not positively or negatively impact on the cost/cost savings or effectiveness; NR=not reported.

**Table A3: Summary of included studies – Treatment ASD**

| **Surname of 1st author, year and**  **brief description of study** | **Evaluation of study** | **Condition^a^, N, Gender^b^, Population^c^, Age** | **Bias** |
| --- | --- | --- | --- |
| **Ashburner, 2016**  Exploration of early intervention parent coaching service delivered via remote technology (Cisco Webex), compared to face-to-face services. Focus of intervention was educating parents about effective approaches for children with ASD (e.g. social narrative and visual schedules). Qualitative study using interviews held with 4 parents, 8 rural service providers and 1 ASD specialist, used content analysis. Benefits and disadvantages of remote technology were discussed. | Clinical effectiveness: NR  Service efficiency: Yes  Economic evaluation: NR  User impact: Yes  Adoption: Yes | ASD  N=12  Mixed  P/C HCP & CYP  2-6y | 5 |
| **Bearss, 2018**  Feasibility trial of parent training (RUBI-PT) program via clinic-to-clinic telehealth. Designed to address a range of behavior and skill deficits in children with ASD and disruptive behaviors using Antecedent-Behavior-Consequence model. Used benchmarking (comparison of new service with findings based on previous clinical trials) to evaluate clinical effectiveness. Attendance was good (93% completion), fidelity high (98%), preliminary efficacy positive - 79% improvement. | Clinical effectiveness: Yes  Service efficiency: NR  Economic evaluation: NR  User impact: Yes  Adoption: Needs research | ASD  N=14  Mixed^m^  P/C & CYP  3-7y | 4 |
| **Clarke, 2018**  Young person had therapy via a voice over internet protocol (VOIP) application with standard desktop or laptop computer hardware and software. After developing therapeutic relationship, was able to attend face-to-face sessions and engage with family and education. | Clinical effectiveness: Yes  Service efficiency: NR  Economic evaluation: NR  User impact: Yes  Adoption: Yes | ASD  N=1  Male  CYP  16y | 4 |
| **Fettig, 2016**  Looked at the effects of e-coaching for a HCP delivering a functional assessment-based intervention in client’s home. The therapist and family met face-to-face for initial training (2hrs) and development of support plan, then a training phase where family implemented plan. E-coaching sessions for HCP were delivered using FaceTime and included training and support. There was a decrease in child’s challenging behaviors. | Clinical effectiveness: Yes  Service efficiency: NR  Economic evaluation: NR  User impact: NR  Adoption: Needs research | ASD  N=2  Female  HCP & CYP  30mth | 4 |
| **Gumundsdottir, 2017**  Description of the development and results of Sunny Starts parent training (behavior intervention involving teaching caregivers methods to increase socio-communicative behavior) delivered via remote technology. Both parent and child showed improvement. | Clinical effectiveness: Yes  Service efficiency: NR  Economic evaluation: NR  User impact: NR  Adoption: Needs research | ASD  N=4  NR  P/C & CYP  3-6y | 4 |
| **Heitzman-Powell, 2014**  Feasibility study to investigate OASIS training for parents of children with ASD. Training included completion of 8 modules online as well as distance coaching sessions via Polycom® videoconferencing delivered at school, community centre or hospital. Training was ABA based and coaching involved discussion of strategies used and feedback on techniques. Parents increased knowledge and implementation of ABA strategies. Over 9000 miles were saved by four families. | Clinical effectiveness: Yes  Service efficiency: Yes  Economic evaluation: NR  User impact: Yes  Adoption: Needs research | ASD  N=7  NR  P/C of CYP  <6y | 4 |
| **Ingersoll, 2015**  Pilot study where parents of children with ASD completed self-directed or therapist-assisted version of ImPACT Online, a novel telehealth-based parent-mediated intervention targeting social communication development. Included 12 self-directed weekly lessons (approx. 80mins/week). In therapist assisted version, parents worked through same training, but also received two 30-minute remote coaching sessions/week via Skype video conferencing. Therapist-assisted parents were more likely to engage with the website (*F*2,24=17.65, *P*<.001) and complete the program (χ21=5.06, *P*=.03) than self-directed. | Clinical effectiveness: NR  Service efficiency: NR  Economic evaluation: NR  User impact: Yes  Adoption: Needs research | ASD  N=28  Mixed  P/C of CYP  27-73mths | 4 |
| **Ingersoll, 2016**  Pilot RCT study comparing effect of self-directed or therapist-assisted version of ImPACT (see Ingersoll, 2015).  Looked at intervention fidelity, self-efficacy, stress, and positive perceptions of child. Both groups showed positive gains, with some slightly better outcomes in the therapist-assisted group, particularly for social skills. | Clinical effectiveness: Yes  Service efficiency: NR  Economic evaluation: NR  User impact: NR  Adoption: Needs research | ASD  N=28  Mixed  P/C of CYP  19-73mths | 3 |
| **Lindgren, 2016**  Evaluation of telehealth costs and acceptability. Compared 3 models of delivery (in-home, clinic-based, home-based telehealth) of parent coaching to implement ABA (FA and FCT training) to treat problem behaviors. All models reduced problem behaviors greater than 90%. Acceptability was high across all groups. Home tele-health was cheapest method of delivery, but clinic led was also cheaper than in-home therapy. | Clinical effectiveness: NO  Service efficiency: Yes  Economic evaluation: Unclear  User impact: Yes  Adoption: Needs research | ASD  N=107  NR  P/C & CYP  1.5-7y | 3 |
| **Little, 2018**  Occupation-based coaching via telehealth on Zoom software (a secure online platform with end-to-end encryption). Focuses on increasing positive interactions between caregivers and child and using learning in everyday routines. Parental efficacy significantly increased post-intervention (p<0.5) and children showed significant improvements in goals set by parents. | Clinical effectiveness: Yes  Service efficiency: NR  Economic evaluation: NR  User impact: NR  Adoption: Needs research | ASD  N=19  NR  P/C & CYP  2-6y | 4 |
| **Little, 2018**  Occupation-based coaching via telehealth using the online videoconferencing platform Zoom software. Included child-parent relationship building, routines, supporting caregivers to use own ideas to advance child. Study assessed parental acceptability and cost-effectiveness of a 12-week telehealth intervention for families of young children with ASD. Parents self-report showed the intervention to be highly acceptable and effective. Cost differences between clinic, at home service, and telehealth models showed exponential savings for families and healthcare providers. | Clinical effectiveness: Yes  Service efficiency: Yes  Economic evaluation: Yes  User impact: Yes  Adoption: Needs research | ASD  N=18  Mixed^m^  P/C of CYP  <6y | 4 |
| **Schieltz, 2018**  Functional communication training (FCT). Sessions were divided into 5 minute blocks, each block was recorded as a separate session. Training continued until child completed a two-step chain twice. The article presents two cases of children with ASD where FCT did not reduce the identified problem behavior. | Clinical effectiveness: No  Service efficiency: NR  Economic evaluation: NR  User impact: NR  Adoption: Needs research | ASD  N=2  Mixed  P/C & CYP  2-6y | 4 |
| **Simacek, 2017**  Parents of children with ASD (n=2) and Rett syndrome (n=1) received functional communication training (FCT) delivered via telehealth. Parents were coached to implement routine and deliver reinforcement each time child used AAC (augmentive and alternative communication) request. All children learned the communication responses that were targeted. Parents were favourable to the telehealth method of delivery. | Clinical effectiveness: Yes  Service efficiency: NR  Economic evaluation: NR  User impact: Yes  Adoption: NR | ASD  N=3  Female  P/C & CYP  <4y | 4 |
| **Suess, 2014**  Parents coached via Skype to conduct functional communication training (FCT). This study evaluates the fidelity of parents conducting the FCT in their own homes following training with a behavior analyst. They found all children substantially reduced problem behaviors and the fidelity was acceptable. | Clinical effectiveness: Yes  Service efficiency: NR  Economic evaluation: NR  User impact: Yes  Adoption: Needs research | ASD  N=3  Male  P/C & CYP  1.5-6y | 4 |
| **Suess, 2016**  Parents coached via skype to conduct functional analysis (1 hour) and communication training (1 hour), they were then further coached as they implemented training for three 15 minute sessions. Problem behavior was reduced by over 65%. | Clinical effectiveness: Yes  Service efficiency: Yes  Economic evaluation: NR  User impact: NR  Adoption: NR | ASD  N=5  Mixed  P/C & CYP  2-7y | 4 |
| **Suess, 2020**  Parents coached via Skype to conduct FCT with differential reinforcement treatments; appropriate behavior training was conducted in three home contexts with minimal or no history of reinforcement for problem behaviours prior to conducting the FCT in the treatment context. Initial findings were comparable to ‘typical’ FCT training with reductions in resurgence happening faster than the traditional FCT. | Clinical effectiveness: Yes  Service efficiency: NR  Economic evaluation: NR  User impact: NR  Adoption: Needs research | ASD  N=4  Male  P/C & CYP  3-6y | 4 |
| **Wainer, 2015**  Telehealth coaching for parents of children with ASD, which combined self-directed internet based information with remote coaching using an imitation intervention. The study examined both parental and child behavior changes and found parents improved their technique and children increased imitation skills. Parents rated the intervention as acceptable, usable and effective. | Clinical effectiveness: Yes  Service efficiency: NR  Economic evaluation: NR  User impact: Yes  Adoption: Needs research | ASD  N=5  NR  P/C & CYP  2-6y | 4 |
| **Wallisch, 2019**  Intervention was reported in Little (2018) paper - this was the linked qualitative evaluation using subsample of patients which explored parents lived experiences of participating in the 12-week telehealth intervention. Parents reported how telehealth fitting in with daily life and led to feelings of empowerment. | Clinical effectiveness: NR  Service efficiency: NR  Economic evaluation: NR  User impact: Yes  Adoption: NR | ASD  N=8  Mixed^m^  P/C & CYP  <7y | 5 |

**NOTES: ^a^Condition:** ASD=autism spectrum disorders, ADHD=attention-deficit/hyperactivity disorder, CD=communication disorders, TD=tic disorder, ID=intellectual disability; **^b^Gender:** Mixed^m^=mixed predominately male, Mixed^F^=mixed predominately female, Mixed=an approximately even split of males and females; **^c^Population:** Participants are defined as those who participated in the intervention. ASU=adult service users, CYP=children and young people, HCP=healthcare professionals, P/C=parents/ caregivers; **^d^Ratings:** YES/NO (was/was not effective/efficient/acceptable/suitable for clinical adoption). Unclear= some outcomes were beneficial, some were not, or increased costs were seen in one area but saved in another. Neutral = did not positively or negatively impact on the cost/cost savings or effectiveness; NR=not reported.

**Table A4: Summary of included studies – Treatment ASD/ADHD**

| **Surname of 1st author, year and**  **brief description of study** | **Evaluation of study** | **Condition^a^, N, Gender^b^, Population^c^, Age** | **Bias** |
| --- | --- | --- | --- |
| **Gillberg, 2017**  Implementation interviews with HCPs regarding a trial using internet based coaching and support for teenagers and young people with ASD and/or ADHD. They reported that the intervention was deemed accessible and distributed quality healthcare services equally, however the barriers related to design and technical issues, staff and organizational issues, and resource allocation. | Clinical effectiveness: NR  Service efficiency: NR  Economic evaluation: NR  User impact: Unclear  Adoption: NR | ADHD/ASD  N=7  NR  HCP | 5 |
| **Sehlin, 2018**  Initial face-to-face meeting followed by Internet-based coaching and support (8 weeks of two weekly chat sessions 30-60mins) using a specially designed chat program. Content analysis showed three themes relating around the decision to participate, taking part in the coaching and the format. Most feedback was favourable with participants appreciating the instructors competence and feeling supported. They did not like the incomplete personal interaction and some experienced technology difficulties. They suggest it is useful as an addition to usual practice. | Clinical effectiveness: NR  Service efficiency: NR  Economic evaluation: NR  User impact: Unclear  Adoption: Add to practice | ADHD/ASD  N=17  Mixed^m^  CYP & ASU  15-32y | 5 |
| **Söderqvist H, 2017**  Paper considered caregivers’ burden after CYP received internet based support and coaching (IBSC). Although caregivers were able to provide functional care as their children transitioned into adulthood, they were worried about their future when they would not be able to provide support. Some caregivers improved, other deteriorated or remained the same post-intervention. | Clinical effectiveness: Unclear  Service efficiency: NR  Economic evaluation: NR  User impact: NR  Adoption: Unclear | ADHD/ASD  N=10  Mixed^F^  P/C & CYP  15-26y | 4 |
| **Pettersson, 2017**  Compared Internet-based cognitive behavioral therapy (iCBT) in a group therapy setting (4-6 people, 3hr, once a week, 10 weeks), with iCBT-S (Internet-based program worked on alone but with opportunity to ask therapists questions via encrypted function in the program) and wait list control. Both used ‘In Focus’ developed by the Swedish company Livanda—Internet Clinic, Ltd., in collaboration with the NPC. They found a significant reduction in ADHD symptoms in the iCBT-S group compared to wait list control (effect size of *d=*1.07), which was maintained at 6-month follow up. However, the dropout rate was high. | Clinical effectiveness: Yes  Service efficiency: NR  Economic evaluation: NR  User impact: Unclear  Adoption: Needs research | ADHD  N=45  Mixed  ASU  18+y | 2 |
| **Shah, 2019**  Group parent training intervention (10 weekly 90 minute sessions) for parents of children with ADHD using the online videoconferencing platform Zoom software. Included psychoeducation, environmental modifications, communication, child-parent relationship building, behavior management, self-care, and parent skills for liaising with teachers. Pilot study exploring feasibility and acceptability of intervention showed parents liked the convenience of telehealth, with cost and time savings. They also reported increased self-competence. Clinicians reported some difficulties with technology such as the audio-video time lag. They report that overall group interventions are feasible and acceptable. | Clinical effectiveness: Yes  Service efficiency: Yes  Economic evaluation: Yes  User impact: Yes  Adoption: Needs research | ADHD  N=11  NR  P/C & HCP | 5 |

**NOTES: ^a^Condition:** ASD=autism spectrum disorders, ADHD=attention-deficit/hyperactivity disorder, CD=communication disorders, TD=tic disorder, ID=intellectual disability; **^b^Gender:** Mixed^m^=mixed predominately male, Mixed^F^=mixed predominately female, Mixed=an approximately even split of males and females;

**^c^Population:** Participants are defined as those who participated in the intervention. ASU=adult service users, CYP=children and young people, HCP=healthcare professionals, P/C=parents/ caregivers; **^d^Ratings:** Neutral=the cost of the intervention or effectiveness is no greater than control, YES/NO (was/was not effective/efficient/acceptable/suitable for clinical adoption). Unclear= some outcomes were beneficial, some were not, or increased costs were seen in one area but saved in another. Neutral = did not positively or negatively impact on the cost/cost savings or effectiveness; NR=not reported.

Table A5: Summary of included studies – Others

| **Surname of 1st author, year and**  **brief description of study** | **Evaluation of study** | **Condition^a^, N, Gender^b^, Population^c^, Age** | **Bias** |
| --- | --- | --- | --- |
| **Bridgman, 2015**  Lidcombe Program via webcam delivery. Paper focuses on the clinical observations of the speech and language pathologist involved in delivering program to families with preschool children who stutter, including participant convenience, attendance, readiness etc as well as clinical boundaries and relationships. | Clinical effectiveness: NR  Service efficiency: Unclear  Economic evaluation: NR  User impact: Unclear  Adoption: Needs research | CD  N=1  Female  HCP of CYP  3-5y | 5 |
| **Bridgman, 2016**  RCT which compared Lidcombe Program via webcam to standard in-clinic (face-to-face) program for families with preschool children who stutter. Looking at the percentage of syllables stuttered at 9-months postrandomization and the number of consultations to complete Stage 1, there were no differences between groups. This suggests that the webcam delivered treatment is as effective and economical as clinic delivered program. | Clinical effectiveness: Unclear  Service efficiency: Unclear  Economic evaluation: Unclear  User impact: Yes  Adoption: Needs research | CD  N=49  Mixed^m^  P/C & CYP  3-5y | 2 |
| **Bridgman, 2018**  Paper focuses on the attitudes and experiences of the speech and language students involved in delivering a pilot telehealth program to families with preschool children who stutter, comparing this to their in-clinic experiences during clinical placement. Students reflected on the challenges of working in telehealth, management of their own anxiety, learning approaches, ease of access, and telehealth considerations. This supports the use of different delivery modes (including telehealth) when training HCPs to enhance students’ competence. | Clinical effectiveness: NR  Service efficiency: NR  Economic evaluation: NR  User impact: Yes  Adoption: Needs research | CD  N=6  NR  HCP | 5 |
| **Carey, 2014**  Phase II non-comparative clinical trial examining the Camperdown Program delivered via webcam for young people who stutter. They found stuttering, in terms of frequency and severity, was reduced at 12- month follow up for approximately half of the young people. Participants reported that the webcam delivery was appealing, reporting increases in satisfaction with fluency and reduced situational avoidance. | Clinical effectiveness: Unclear  Service efficiency: Yes  Economic evaluation: NR  User impact: Yes  Adoption: Needs research | CD  N=16  Male  CYP  12-17y | 4 |
| **Ferdinands.2019**  Data was drawn from Bridgman, 2016, looking at parent satisfaction with child fluency, percentage of syllables stuttered and parental rated severity in clinic and telehealth delivered Lidcombe programs. Looking at baseline and 9/18 month follow-ups, they found parental satisfaction generally resulted in a decrease in stuttering severity, but conclude this could not be measured on an ordinal scale. | Clinical effectiveness: Yes  Service efficiency: NR  Economic evaluation: NR  User impact: NR  Adoption: Needs research | CD  N=49  Mixed^m^  P/C of C  3-5y | 2 |
| **Jahromi, 2018**  Study assessed satisfaction of tele-speech therapy via Skype videoconferencing for people who stutter. Most patients were study with tele-speech therapy, although the speed of Internet was a challenge. Although the authors recommend the program, they suggest that improving Internet connectivity would improve the success of telehealth programs. | Clinical effectiveness: NR  Service efficiency: Yes  Economic evaluation: Yes  User impact: Unclear  Adoption: Yes | CD  N=30  Mixed^m^  CYP & ASU  14-39y | 5 |
| **O'Brian, 2014**  Phase I trial of the Lidcombe Program exploring the efficacy, practicality and viability of delivering the program via webcam. At 6-months follow up, all children were below 1% syllables stuttered. Parents found the webcam delivery acceptable, practical and viable. The authors recommend further clinical trials and qualitative research. | Clinical effectiveness: Yes  Service efficiency: NR  Economic evaluation: NR  User impact: Yes  Adoption: Needs research | CD  N=3  Mixed^m^  P/C of CYP  3-4y | 4 |
| **Ricketts, 2016a**  Examined the preliminary efficacy, feasibility, and acceptability of the Comprehensive Behavioral Intervention for Tics Voice over Internet Protocol (CBIT-VoIP) which included HRT, psychoeducation about tics, self-monitoring of tics, function-based assessment and intervention, and diaphragmatic breathing and progressive muscle relaxation. Pilot study involving 8 weekly CBIT sessions showed nearly 30% reduction in clinician-rated tic severity. Satisfaction ratings from HCPs, child and parent were high. CBIT was considered feasible to implement via VoIP, although further testing is recommended. | Clinical effectiveness: Yes  Service efficiency: NR  Economic evaluation: NR  User impact: Yes  Adoption: Unclear | TD  N=10  Mixed^m^  CYP  10-14y | 4 |
| **Ricketts,2016b**  Study 1: Used Skype to administer YGTSS. Another researcher in the home acted as second rater for control.  Study 2: Randomised, waitlist-controlled pilot trial examined the efficacy, feasibility and acceptability of the Comprehensive Behavioral Intervention for Tics (CBIT), involving weekly sessions via VoIP providing therapist support and parent completion of workbook and homework assignments. One-third of children in the CBIT-VoIP were considered treatment responders. Significantly greater reductions in both clinician and parent rated tic sevirtiy were found in the CBIT-VoIP groups compared to controls. Satisfaction was high and generally feasible to implement albeit with minor audio and visual difficulties. The authors suggest modifications to enhance treatment delivery which can be tested prior to widespread adoption. | Clinical effectiveness: Yes  Service efficiency: NR  Economic evaluation: NR  User impact: Unclear  Adoption: Needs research | TD  N=20  Mixed^m^  CYP  8-16y | 2 |
| **Merrill, 2017**  Ohio’s Telepsychiatry Project for Intellectual Disability enables individuals with intellectual disability and a co-occurring mental illness to access psychiatric care via telehealth. Patients from 68 counties in Ohio are involved in the project. The project has been running since 2012 and has led to improved access to care, reduced hospitalization, and reduced travel and support costs. It has also resulted in greater communication with patients. | Clinical effectiveness: Yes  Service efficiency: Yes  Economic evaluation: Yes  User impact: Yes  Adoption: Yes | ID  N=>1000  NR  NR | 5 |

**NOTES: ^a^Condition:** ASD=autism spectrum disorders, ADHD=attention-deficit/hyperactivity disorder, CD=communication disorders, TD=tic disorder, ID=intellectual disability; **^b^Gender:** Mixed^m^=mixed predominately male, Mixed^F^=mixed predominately female, Mixed=an approximately even split of males and females;

**^c^Population:** Participants are defined as those who participated in the intervention. ASU=adult service users, CYP=children and young people, HCP=healthcare professionals, P/C=parents/ caregivers; **^d^Ratings:** Neutral=the cost of the intervention or effectiveness is no greater than control, YES/NO (was/was not effective/efficient/acceptable/suitable for clinical adoption). Unclear= some outcomes were beneficial, some were not, or increased costs were seen in one area but saved in another. Neutral = did not positively or negatively impact on the cost/cost savings or effectiveness; NR not reported.
